# Multi-objective integrated optimization study of prefabricated building projects introducing sustainable levels

**DOI:** 10.1038/s41598-023-29881-6

**Published:** 2023-02-17

**Authors:** Junlong Peng, Yue Feng, Qi Zhang, Xiangjun Liu

**Affiliations:** School of Traffic and Transportation Engineering, Changsha University of Science and Technology, Changsha, 410114 China

**Keywords:** Engineering, Civil engineering

## Abstract

As construction becomes greener, people have higher and higher requirements for engineering project management, which makes it necessary to deeply study the comprehensive optimization of schedule, cost and sustainability level. Adhering to the concept of low carbon and green, the article takes carbon emission factor into the total cost of building construction and improves the traditional cost objective of engineering projects; then quantitatively analyzes the economic, environmental and social impacts of assembled buildings from the perspective of sustainability, and introduces the sustainability objective into the traditional duration-cost problem study, taking the duration of each job in the double code arrow diagram as the independent variable to construct the duration -cost-sustainability level multi-objective optimization model. In order to solve the type effectively, a series of Pareto optimal solutions are obtained using the NSGA-II algorithm, and the efficacy coefficient method is used for program decision making. The results show that the Pareto solution set can provide effective support for the project manager’s decision making, and the NSGA-II algorithm is effective in solving the multi-objective optimization model.

## Introduction

**Figure 1:**
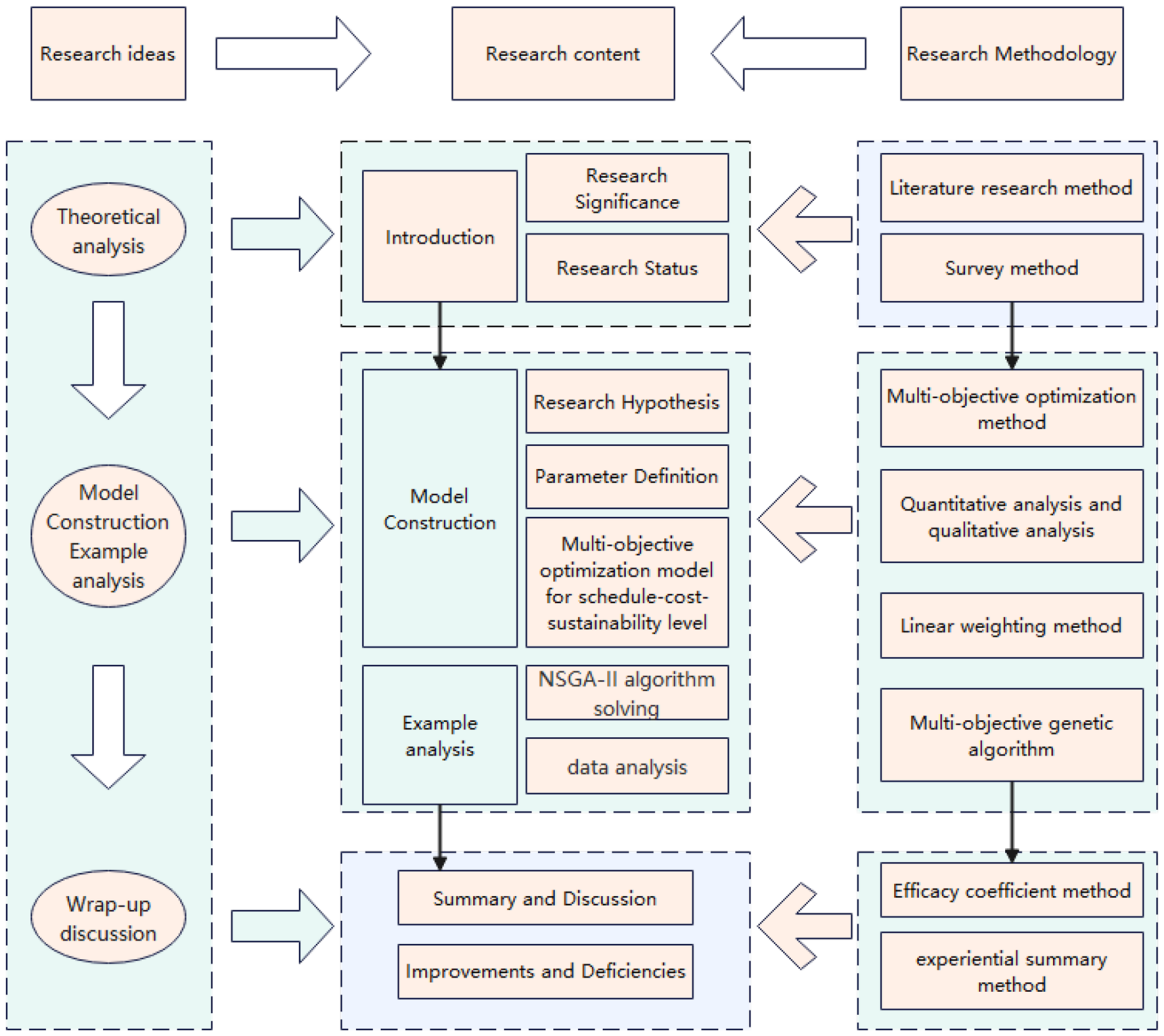
Technology roadmap.

Sustainable development is the result of mankind’s reflection on industrial civilization and a rational choice made by mankind to overcome a series of environmental, economic and social problems, especially global environmental pollution and ecological damage, and the imbalance in the relationship between them^1^. With the development of science and technology and the rapid growth of the global economy, the problem of contradiction between development and environment is becoming more and more acute. The earth has long suffered from the impact of human activities, and the earth’s temperature has continued to rise due to the greenhouse effect, becoming a major threat to human survival, so countries around the world are actively taking measures to seek effective tricks to deal with the increasingly serious environmental problems. The currently advocated green low-carbon economic model with low pollution and low energy consumption responds to the development requirements of the times. According to the UNEP Global Status of Buildings and Construction 2020 report, buildings and construction account for 35$\%$ of global final energy use and 38$\%$ of energy-related CO$_2$ emissions in 2019. The construction industry is a major contributor to global energy consumption and carbon emissions, and the implementation of sustainable concept buildings brings environmental, economic and social benefits at all levels. As the main type of industrialized system building, assembled buildings are an effective way to drive technological progress. However, at the present stage, assembled buildings have encountered various bottlenecks in practice, and the industry market is currently facing serious homogeneous competition, poor quality of workers’ skills, and unsound regulatory mechanisms, which have restricted the better development of the industry. There is a lack of scientific, objective and comprehensive research on the sustainability of assembled buildings, and a lack of comprehensive exploration of the sustainability of assembled buildings from various perspectives such as resources, economy, society and environment. This thesis focuses on researching the sustainability of construction enterprises and introducing sustainability goals into traditional engineering project management to improve the sustainability of assembled buildings. The technical roadmap of this paper is shown in Fig. 1.

## Literature review

Multi-objective optimization problems and optimization model building have been proposed as early as the nineteenth century, and the study of traditional multi-objective optimization problems first started with two-objective optimization of duration and cost. The traditional multi-objective first focused on modeling through the duration-cost relationship, and the relationship between the two was generally considered as a convex curve.Harvey^2^ was the first to analyze the relationship between duration and cost and proposed the duration-cost balance problem. Babu^3^ proposed to apply the linear programming model in a multi-objective integrated schedule-cost optimization study. Hazir O^4^ constructed three robust optimization models for discrete duration cost optimization problems, which laid a good foundation for the study of robust optimization models for duration cost of discrete engineering projects.With the in-depth study of project management, the tri-objective optimization problems for projects are mainly focused on cost, schedule and quality. Pollack-Johnson^5^ used hierarchical analysis to calculate the quality level of each process and finally proposed an integer linear programming model. ELRrayes k^6^ made different combinations of construction methods, number of workers and processing modes that affect these three objectives under a multi-objective integrated optimization model considering time, cost and quality, which resulted in different process construction solutions. Azaron^7^ developed a multi-stage, multi-objective integrated optimization model for duration, cost, and quality in a stochastic network planning diagram environment.With the progress of science and technology, more scientific theories are applied to the multi-objective study of project management. Ali^8^ made more relaxed linear assumptions in the duration-cost-quality optimization model, thus minimizing the effects of duration, cost and quality variations and making it closer to engineering practice.Zhang and Zue^9^ established a fuzzy equilibrium with process duration as the decision variable by combining fuzzy theory to analyze the relationship between the three objective functions of time, cost and quality optimization model, and proposed a genetic immune particle swarm optimization algorithm based on particle swarm algorithm. Shahsavari^10^ constructed a duration-cost-quality multi-objective integrated optimization model based on quantification of quality objectives using linguistic variables and fuzzy theory, solved it using genetic algorithms. Zhuo^11^ used a particle swarm algorithm to solve a duration-cost-quality. Xingguan Chen^12^ obtained a project duration-cost-quality prediction model by Monte Carlo simulation based on earned value theory. Ning Lu^13^ proposed the earned value method for construction safety management, which provides a quantitative approach to safety for subsequent scholars’ research by quantifying safety gains. In recent years, more and more scholars have started to include energy saving and environmental protection into the consideration of engineering management, and the research on the tri-objective optimization problem of some engineering projects has focused on the comprehensive optimization of the three objectives of schedule, cost and energy saving and environmental protection, while tending to use artificial intelligence algorithms to solve their models. Li Wen^14^ optimized the project cost and time by particle swarm optimization algorithm based and combined with optimization techniques of BIM construction engineering. Xin Zou^15^ constructed a framework for measuring environmental performance assessment of buildings through which adverse environmental impacts on construction sites were minimized. Fan^16^ studied a multi-objective optimization model for building envelope retrofitting schemes to retrofit existing buildings for energy efficiency. Fabrizio Ascione^17^ constructs Harlequin, a new integrated framework for multi-objective building energy design, for multi-stage and multi-objective design optimization of building energy consumption. Junqing Li^18^ proposed the use of genetic algorithm-based BIM models to explore building energy efficiency methods. Caleb Debrah^19^ proposed the application of artificial intelligence in the field of green building to improve the sustainability of the construction industry. Liang^20^ achieved multi-objective optimization of the construction process at the micro level and used an ant colony algorithm to optimize the cost-duration-carbon model of the assembly building process to obtain different combinations of process execution modes.Research on four-objective optimization problems in engineering projects focuses on the integrated optimization of cost, schedule, quality, and safety. duc Hoc Tran^21^ helps project decision makers to successfully complete projects on time and reduce risks by using adaptive multi-objective differential evolutionary algorithms to solve schedule-cost-safety optimization models for engineering projects. Zhao Dong^22^ integrates genetic algorithms into a BIM platform and introduces methods such as virtual construction and collision checking to deal with more complex multi-objective problems of schedule, cost, quality, and safety. Yong Xiang^23^ used multi-objective particle swarm optimization algorithm to solve the multi-objective equilibrium optimization model of schedule, quality, safety and environment of engineering projects.

At present, research on sustainable aspects of assembled building projects is mainly reflected in the following areas: Sustainability evaluation analysis of assembled buildings from a life cycle perspective. Tomonari Yashiro^24^ introduced the concepts of innovative technology, production system, total life cycle management, and supply chain management for assembled buildings based on the study of technical, economic, social aspects to provide ideas for future research on assembled buildings. Onat^25^ evaluated and quantified the sustainability of U.S. homes by using the LCSA framework, and the study found that the greatest environmental impact of buildings was on electricity use. Nikola Maodus^26^ analyzed the performance and whole life cycle assessment of assembly building materials from an economic and environmental point of view as a way to develop new energy-efficient, environmentally friendly, and low-cost assembly building materials. Jingjing Wang^27^ used BIM to perform a full life-cycle evaluation of demolition waste from sustainable buildings and to assess their effective reuse capacity. Dhaif^28^ studied the energy performance and life-cycle costs of prefabricated buildings using the method of operational energy use dynamic energy simulation tool.

Supply chain management perspective for sustainability analysis of assembled buildings. Gunasekaran^29^ proposed business sustainability in manufacturing and argued that supply chain management is a key driver for the establishment of sustainable competitive advantage for companies. Hu^30^ conducted a scientific evaluation study on the sustainability of assembled buildings from the perspective of green supply chain stakeholders, using three perspectives: designers, manufacturers, and suppliers of the assembled building supply chain. Yue Zhai^31^ proposed a buffer space hedging coordination mechanism to solve the hedging problem in the supply chain management of assembled buildings, and further exploit the advantages of assembled buildings by improving the supply chain management. Yinghui Song^32^ proposed a supplier evaluation index system based on equation modeling and intuitive fuzzy hierarchical analysis for the construction of assembly buildings, and the study showed that quality has the most significant impact on supplier selection.

Through comprehensive analysis of the literature, the following thoughts and summaries are given for the research on multi-objective optimization of engineering projects. (1) From the perspective of optimization model, the objectives considered for engineering project management are becoming more and more perfect and rich, and the assumptions and model solving methods in model establishment also tend to be mature and perfect. From the initial study of dual-objective optimization of duration-cost, it gradually developed into multi-objective optimization of duration-cost-quality, duration-cost-quality-safety, etc., and achieved good results in practical application. However, most of the studies have failed to fully consider sustainable factors such as environmental protection and social benefits, and the optimization models established need to be further improved. (2) From the perspective of research on the sustainability of assembled buildings, most scholars have focused their research on the theoretical study of the development mode of the assembled building industry, exploring the development of the assembled industry from the analysis and evaluation of the industry and the technical perspective. Mainly focusing on the overall sustainability construction of the whole construction industry, most of the research on it stays in qualitative analysis, rarely from the microscopic perspective of assembly building project management, and there is less research on the dynamics of its assembly building management. Therefore, this study introduces the sustainability level into multi-objective optimization management to quantitatively analyze the economic, environmental and social impacts of assembled buildings from the sustainability perspective, followed by adding the sustainability objectives into the traditional multi-objective integrated optimization of assembled buildings, and finally solving the duration-cost-sustainability model through algorithms to provide a reference for the sustainable development of assembled buildings.

## Materials and methods

### Multi-objective optimization problems for engineering projects

**Figure 2:**
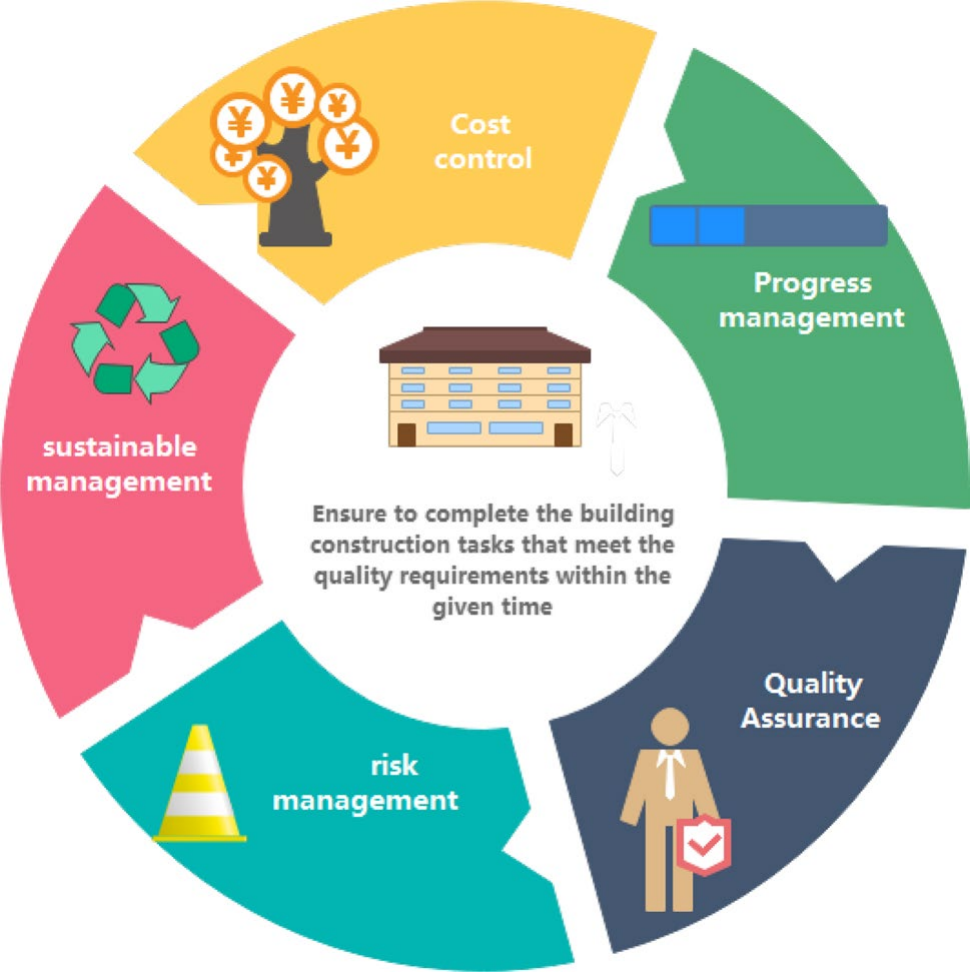
Engineering project management process.

Engineering project management is to plan, coordinate and control the whole process of project implementation under certain resource constraints with the building as the construction target, and to ensure the completion of the building construction task in accordance with the quality regulations within the established time. The engineering project management process is shown in Fig. 2. In the actual construction process, the relationship before each goal is both antagonistic and unified. As the relationship between schedule, cost and sustainability level studied in the article, there are contradictions and opposites among the three major objectives of project schedule, cost and sustainability, and such opposites are concentrated in the constraint relationship and mutual influence among them. The constraints are expressed in the shortening of the duration of the project will increase the cost, the impact on the environment will increase, and the sustainable level of the project will also be reduced in the process of rushing; if the cost is reduced, although the economic benefits of the project can be increased to a certain extent, the sustainable level will increase, but it will cause the duration of the project to be extended. There is also a unified aspect between the three major goals of project schedule, cost and sustainability, and this unified relationship is concentrated in the promotion relationship and balance relationship among them. The unified aspect is that increasing investment can speed up the construction schedule, while the environmental impact can be appropriately reduced, and the increased investment in the early stage can reduce the maintenance cost of the later project, the economic benefits of the whole life cycle of the project can be improved, and the sustainable level of the project can be increased; appropriately improving the sustainable level of the project, although it will cause an increase in the construction period, can save the operating cost of the project after it is put into use, and Improve the social benefits, so as to obtain better economic benefits of investment. Therefore, in the process of project management, investment, schedule and sustainability should be considered as a system, repeatedly coordinated and balanced, and the construction should be reasonably organized, so as to achieve the optimum of the whole target system.

### Multi-objective optimization theory

A multi-objective optimization strategy can optimize multiple objectives. The relationship between multiple objectives is mutually constraining, interacting and cannot be optimal at the same time. In order to achieve the best tradeoff among all objectives, the Pareto optimal solution set gives a reasonable explanation. In general, the relevant definition of multi-objective optimization is as follows:

1. Set the objective function*F*(*x*); The given optimization problem can be formulated as follows:
  1 $$\begin{aligned} \begin{array}{l} \min F(x) = {f_1}(x),{f_2}(x), \ldots ,{f_k}(x)\\ s.t.{g_i}(x) \le 0,i = 1,2, \ldots ,m\\ {h_j}(x) = 0,j = 1,2, \ldots ,p \end{array} \end{aligned}$$
  where,$x = ({x_1},{x_2}, \ldots ,{x_n})$is the *n* dimensional decision vector,${f_i}:{R^{\rm{{n}}}} \rightarrow R,i = 1,2, \ldots ,k$ is the objective function, and${g_i},{f_j}:{R^{\rm{{n}}}} \rightarrow R,i = 1,2, \ldots ,p$ are the inequality constraint and the equation constraint, respectively.
2. Pareto-dominant^33^;Assumptions$\overrightarrow{m} = ({m_1},{m_2}, \ldots ,{m_k})$ and $\overrightarrow{n} = ({n_1},{n_2}, \ldots ,{n_k})$ also $m < n$, if and only if:
  2 $$\begin{aligned} \begin{array}{l} \forall \text {i} \in [1, \text {k}],\left[ \text {f}\left( \text {m}_{\text {i}}\right) \le \text {f}\left( \text {n}_{\text {i}}\right) \right] \wedge \left[ \text {i} \ni [1, \text {k}], \text {f}\left( \text {n}_{\text {i}}\right) \right] \end{array} \end{aligned}$$
3. Pareto optimality; assuming that $\overrightarrow{m}$ is Pareto optimal, if and only if:
  3 $$\begin{aligned} \begin{array}{l} \not \exists \textbf{n} \in M \text{ s.t. } F(\textbf{n})<F(\textbf{m}) \end{array} \end{aligned}$$
4. Pareto optimal solution set:
  4 $$\begin{aligned} \begin{array}{l} \not \exists \textbf{n} \in M \text{ s.t. } F(\textbf{n})<F(\textbf{m})S_{i}:=\{m, n \in M \mid \exists F(n)>F(m)\} \end{array} \end{aligned}$$
5. Pareto optimal frontier:
  5 $$\begin{aligned} \begin{array}{l} S_{i}:=\left\{ F(m) \mid m \in S_{i}\right\} \end{array} \end{aligned}$$

## Multi-objective optimization model

### Research hypothesis

1. The construction sequence of the project remains unchanged, without considering the stoppage caused by force majeure factors such as weather and policies.
2. The construction results of the project have achieved the expected use and service effect, and the use effect meets the planning and design requirements. On this basis, the economic evaluation of the sustainable aspects of its project is transformed into a cost analysis.
3. The carbon emission sources of the project include only the workers’ breath, materials and machinery energy consumption during the construction phase, excluding the carbon emission due to other factors.
4. The environmental level of an engineering project has a non-linear relationship with the process duration. If the duration of the project process is shortened, the pollution to the environment will be aggravated by the rush work, which will have a greater impact on the environmental level; if the duration of the project process is too long, the impact on the environment will continue to increase with the extension of the construction period. The overall environmental level shows a U-shaped trend of decreasing and then increasing with the increase of process duration.
5. The level of social responsibility of engineering projects has a non-linear relationship with the duration of process activities. If the project process duration is shortened, the project builder will increase the investment in human and material resources, including the increase in the number of jobs; if the project process duration is too long, the project will reduce the number of jobs due to cost saving. The overall level of social responsibility shows a “U” shaped trend of increasing and then decreasing with the increase of process time.

### Parameter definition

Parameters are defined in Table 1.

**Table 1:** Parameter definition.

Parameters	Parameter definition	Parameters	Parameter definition
*T*	Total project duration	${\omega _2}$	Environmental goal weighting factor
*I*	The set of processes on a critical line in the network plan	${\omega _3}$	Social goal weighting factor
${t_{si}}$	Minimum duration of process i	${H_i}$	Total workdays of workers to complete process i
${t_i}$	Actual duration of process i	${C_{hi}}$	Per capita respiratory carbon emission factor
${t_{li}}$	Maximum duration of process i	${M_i}$	Consumption of the first material to complete process i
${t_{ni}}$	Normal duration of process i	${C_{mj}}$	Carbon emission factor of the jth material
${C_Z}$	Direct cost of process i	${U_i}$	Completion of the first energy consumption to complete process i
${C_J}$	Indirect costs of process i	${C_{sk}}$	Carbon emission factor of the kth energy source
${C_B}$	Variable cost of early or late completion of works	${E_A}$	Total emissions of all air pollutants
${C_T}$	Carbon cost of process i	${E_D}$	Total emissions of all solid waste
${C_{ni}}$	Direct costs for the normal duration of process i	${E_W}$	Total volume of all sewage discharges
${\alpha _{1i}}$	Incremental marginal cost factor	$E{I_i}$	Environmental level index of process i
${\alpha _{2i}}$	Overhead costs per day for process i	$E{I_{i\max }}$	Limit values of environmental level of the process
${\alpha _{3i}}$	Variable cost factor	$W{I_i}$	Number of jobs provided by completed processes
${\alpha _{4i}}$	Carbon tax rate for construction industry	*K*	Sustainability level index
${\omega _1}$	Economic target weighting factor	${C_{Ti}}$	Carbon cost of process i

### Construction time target

The duration of this paper is the actual progress of the construction phase of the project determined based on the network plan. The time required for the most time-consuming line in the network plan is the duration of this project, i.e. the sum of the work durations on the critical lines is the total project duration. The schedule optimization model is as follows:

6 $$\begin{aligned} \begin{array}{l} \min T = \sum \nolimits _{i \in l} {{t_i}} \\ s.t. \quad {t_{si}} \le {t_i} \le {t_{li}} \end{array} \end{aligned}$$

### Cost objectives

In this paper, the total project cost is defined as consisting of direct cost ${C_Z}$, indirect cost ${C_J}$, variable cost ${C_B}$, and carbon emission cost ${C_T}$. Direct costs include the costs directly invested in the production of the project such as labor, materials and machinery; indirect costs include the costs indirectly invested in the construction of the project such as management fees and staff travel; variable costs include the penalty costs caused by the delay in the construction period or the incentive costs generated by the early construction period. Carbon emission cost refers to the cost of implementing measures to avoid potential impact on the environment during the construction of engineering projects, which is mainly the cost arising from carbon emission. By taxing carbon emissions, the environment can be protected and attention can be drawn to it. The cost optimization model is as follows:

7 $$\begin{aligned} \left\{ \begin{array}{l} \min C = {C_Z} + {C_J} + {C_B} + {C_T}\\ {C_Z} = \sum \nolimits _{i = 1}^l {\left[ {{C_{ni}} + {\alpha _{1i}}{{({t_i} - {t_{ni}})}^2}} \right] } \\ {C_J} = \sum \nolimits _{i = 1}^l {({\alpha _{2i}}{t_i})} \\ {C_B} = {\alpha _{3i}}(T - {T_r})\\ {C_T} = \sum \nolimits _{i = 1}^l {{\alpha _{4i}}({H_i}{C_{hi}} + \sum \nolimits _{j = 1}^n {{M_i}{C_{mj}} + \sum \nolimits _{k = 1}^m {{U_i}{C_{sk}})} = \sum \nolimits _{i = 1}^l {{C_{Ti}}} } } \\ s.t. \quad \left\{ \begin{array}{l} {t_{si}} \le {t_i} \le {t_{li}}\\ 0 \le T \le {T_r} \end{array} \right. \end{array} \right. \end{aligned}$$

### Sustainability goals

This paper quantifies the sustainability objectives from three dimensions of sustainable development: economic, environmental and social, and constructs a multi-objective optimization model for the sustainable level of assembled buildings. The economic sustainability of the assembled building studied in this thesis refers to the minimum total cost of assembled building construction, i.e., to maximize the economic benefits of the building project by trying to achieve the expected use and service effects with the minimum expenditure, without analyzing the optimal cost effects of the whole life cycle of the assembled building. Based on this, the total cost minimization model established in the previous section is the economically sustainable model for assembled buildings. The economic model is as follows:

8 $$\begin{aligned} \begin{array}{l} \text {f}_{1}=\text {min} \text {C}=\text {C}_{\text {Z}}+\text {C}_{\text {J}}+\text {C}_{\text {B}}+\text {C}_{\text {T}} \end{array} \end{aligned}$$

Environmental sustainability of assembled buildings refers to the minimal impact on the environment generated during the construction of assembled buildings. According to the whole-life assessment method, the environmental impacts of engineering structures include the adverse effects of air pollutants, sewage and solid waste emissions^34^ on human health, ecosystems, global climate, and population habitats. Drawing on previous literature^35^ to quantify environmental objectives, environmental emissions are the product of emission factors and the number of process activities, and the model with the smallest environmental level index is constructed as the environmental sustainability model of the assembly building. The environmental model is as follows:

9 $$\begin{aligned} \begin{array}{l} \left\{ \begin{array}{l} EI_{i}=\frac{E_{A}}{E_{A \max }}+\frac{E_{D}}{E_{D \max }}+\frac{E_{W}}{E_{W \max }} \\ E I=\sum _{i=1}^{l} E I_{i} \\ f_{2}=\min E=\sum _{i=1}^{l} E I_{i}\left( t_{i}-t_{n i}\right) ^{2} \end{array}\right. \end{array} \end{aligned}$$

Social sustainability of assembled construction refers to the project’s contribution to local sustainable development by creating social benefits. In this paper, we take the maximum social responsibility as the goal of measuring social benefits, and stipulate that social responsibility consists of the number of jobs created by the completion of the project to the society, such as the number of workers who complete each process with the duration, when the process duration is short, the project will need more workers due to the rush; conversely, the builder will reduce the number of jobs appropriately due to the long duration to save costs. The relationship between social responsibility level index and process duration is non-linear and shows the characteristics of quadratic curve by fitting, therefore, the model with the largest social responsibility level index is constructed as the social sustainability model of assembly building. The social responsibility level model is as follows:

10 $$\begin{aligned} {f_3} = \max W = \sum \nolimits _{i = 1}^l {W{I_i}{{({t_i} - {t_{ni}})}^2}} \end{aligned}$$

Based on the above analysis, the sustainable objective function in this paper includes economic objective function, environmental objective function and social responsibility objective function. Firstly, the objective function values under each objective function are dimensionless by Eq. (11), secondly, each objective weight is set, and the above multi-objective function is converted into a single objective function by Eq. (12) using linear weighting, and since the minimum value is required for the economic objective function and environmental objective function, and the maximum value is required for the social responsibility objective function, $f_{1}^{\prime }$,$f_{2}^{\prime }$ is taken as negative value and $f_{3}^{\prime }$ is taken as positive value. The sustainability level optimization model is as follows:

11 $$\begin{aligned}{} & {} \begin{array}{l} \text {f}_{\text {i}}^{\prime }=\frac{\text {f}_{\text {i}}-\text {f}_{\max }}{\text {f}_{\max }-\text {f}_{\min }}, \quad \text {i}=1,2,3 \end{array} \end{aligned}$$

12 $$\begin{aligned}{} & {} \begin{array}{l} \begin{array}{c} {\text {maxK}}=w_{1} \cdot \text {f}_{1}^{\prime }+w_{2} \cdot \text {f}_{2}^{\prime }+w_{3} \cdot \text {f}_{3}^{\prime } \\ \text{ s.t } \left\{ \begin{array}{c} \omega _{1} \ge 0, \quad \omega _{2} \ge 0, \quad \omega _{3} \ge 0 \\ \omega _{1}+\omega _{2}+w_{3}=1 \end{array}\right. \end{array} \end{array} \end{aligned}$$

### Schedule-cost-sustainability level multi-objective optimization model

13 $$\begin{aligned} \begin{array}{l} \left\{ \begin{array}{l} f(T,C,K) = \left\{ \begin{array}{l} \min T = \sum \nolimits _{i \in l} {{t_i}} \\ \min C = {C_Z} + {C_J} + {C_B} + {C_T}\\ \max K = {\omega _1} \cdot {{f'}_1} + {\omega _2} \cdot {{f'}_2} + {\omega _3} \cdot {{f'}_3} \end{array} \right. \\ s.t\left\{ \begin{array}{l} {t_{si}} \le {t_i} \le {t_{li}}\\ 0 \le E{I_i} \le E{I_{i\max }}\\ 0 \le T \le {T_r}\\ {\omega _1} + {\omega _2} + {\omega _3} = 1\\ {\omega _1} \ge 0,{\omega _2} \ge 0,{\omega _3} \ge 0 \end{array} \right. \end{array} \right. \end{array} \end{aligned}$$

## NSGA-II algorithm

**Figure 3:**
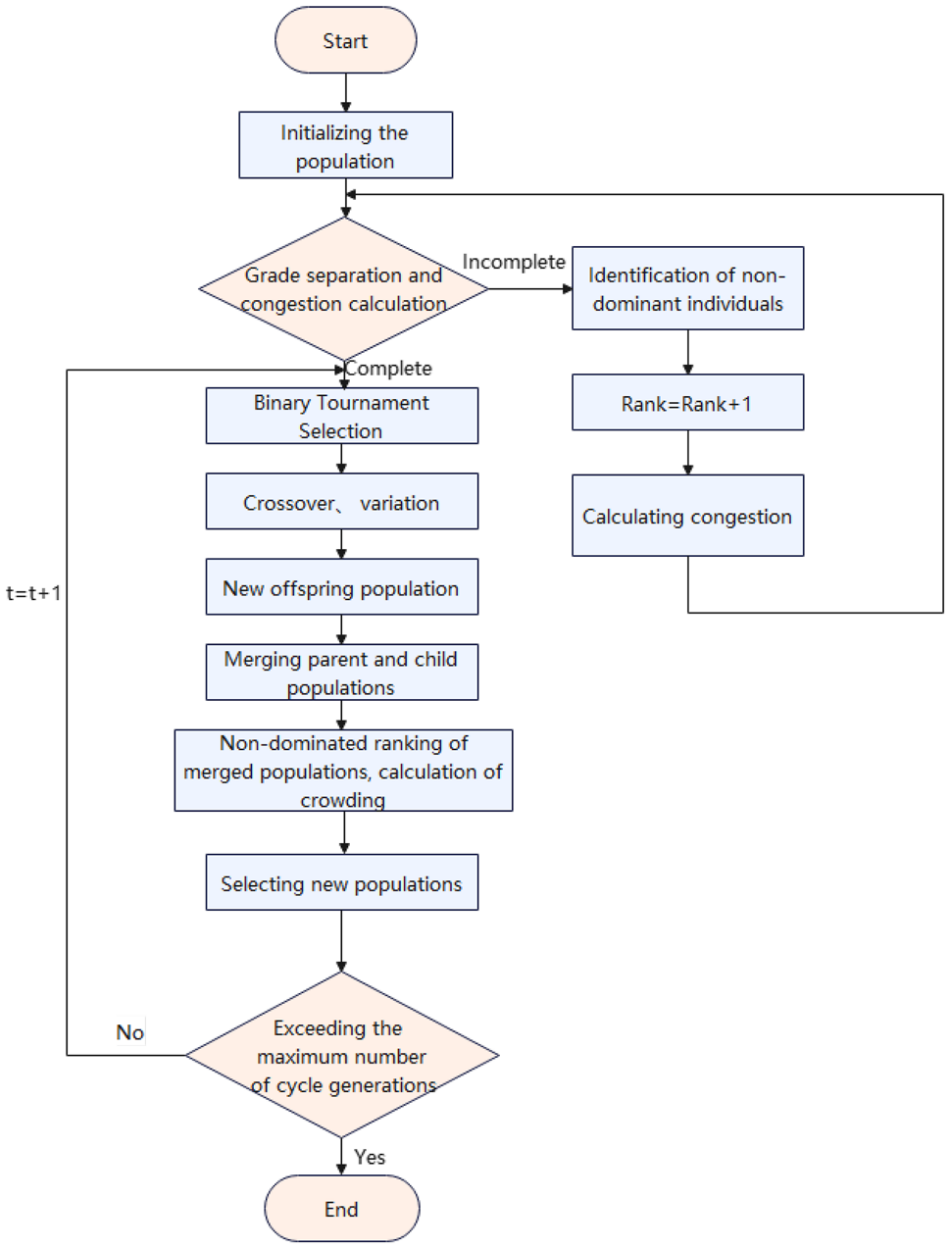
Basic flow chart of NSGA-II algorithm.

Although the research on multi-objective genetic algorithms has made great progress over the years, it still has shortcomings, firstly, the computational complexity of non-dominated sorting makes NSGA computationally expensive in large scale population; secondly, the lack of elite strategy leads to the inability to converge to the global optimal solution; although the algorithm has made progress in dynamic scaling of shared parameters, it still needs to specify shared parameters to obtain diverse solutions. With the NSGA-II algorithm proposed by Deb in 2000, the algorithm effectively solves the above-mentioned shortcomings of NSGA. The operation process of NSGA-II algorithm is based on the classical genetic algorithm operating selection, crossover, and variation, and the targeted improvements mainly include the introduction of the elite strategy, the proposed fast non-dominated sorting method based on grading and the crowding degree and crowding degree comparison operator, which increases the sampling space, reduces the complexity of the algorithm, and eliminates the need to artificially formulate shared parameters^36^. Due to the strong advantages of NSGA-II, the NSGA-II algorithm is chosen to solve the duration-cost-sustainability level multi-objective optimization model in this paper. Specifically, the NSGA-II algorithm in this paper includes the following steps: The basic flow of the NSGA-II algorithm is shown in Fig. 3.

Step 1: Initialize the population and chromosome coding Let *N* be the number of individuals in the population, randomly generate the initial population of size *N*. Each randomly generated chromosome needs to be compared with all the previously generated individuals, and if they are not the same, they are added to the initial population, if they are the same, they are discarded to ensure the population diversity.

Step 2: Hierarchical separation and crowding calculation, The population was ranked in non-dominance hierarchy, and the higher the hierarchy, the higher the individual fitness. Crowding distance is used to measure the fitness of individuals in the same non-dominated hierarchy. In order to maintain the diversity of the population, we need an operator to compare crowding to ensure that the algorithm converges to a uniformly distributed Pareto surface. The crowding distance of a bounded individual is set and the crowding degree of other individuals is calculated by the following formula.

14 $$\begin{aligned} \begin{array}{l} \delta _{d}(l)=\sum _{s=1}^{g} \frac{\left| E_{s}(1+l)-E_{s}(1-l)\right| }{E_{s}^{\max }-E_{s}^{\min }}, 1 \in \{2,3 \cdots , n-1\} \end{array} \end{aligned}$$

Equation (14) The number of objective functions is denoted as *g*, the rank of the *l*th individual is denoted as *d*, the number of individuals with rank *d* is denoted as *d*; the value of the *S*th objective function is denoted as ${E_S}$ ; the crowding distance of the *l*th individual is denoted as ${\delta _d}(l)$ , the maximum value of the *S*th objective function is denoted as $E_s^{\max }$, and the minimum value of the *S*th objective function is denoted as $E_s^{\min }$ .

Step 3: Selection,The selection of high-quality individuals in the population is performed by the classical binary tournament selection method. First, the solutions in the generated population that do not satisfy their own performance constraints are eliminated, and then the binary tournament selection method is used for the remaining solutions. According to the calculation results of crowding degree and ranking, all individuals of the population species will be given two attributes, non-dominated ranking order and crowding distance, and the non-dominated ranking level will be compared, and the individual with small ranking value will be selected if the levels are unequal; if the levels are equal, the crowding degree will be compared and the one with larger crowding degree will be selected. The process is repeated, and two parent individuals are selected for the next step to obtain crossover variation.

Step 4: Crossover, mutation Using the crossover mutation method to solve, each crossover mutation produces two offspring and cycles the crossover mutation process until a new population ${Q_t}$ with population size *N* is produced.

Step 5: Population Merger, Merge parent population ${P_t}$ and offspring population ${Q_t}$ into a new population ${H_t}$ with population size 2*N*.

Step 6: Non-dominance ranking and crowding calculation,Perform non-dominance ranking and crowding calculation on population ${H_t}$ to produce a new population ${P_{t + 1}}$. Remove the same chromosomes in the new population, perform non-dominance ranking and crowding calculation, and select*N* individuals with higher fitness to form a new population ${P_{t + 1}}$.

Step 7: Cycle number judgment

## Example analysis

### Example introduction

**Figure 4:**
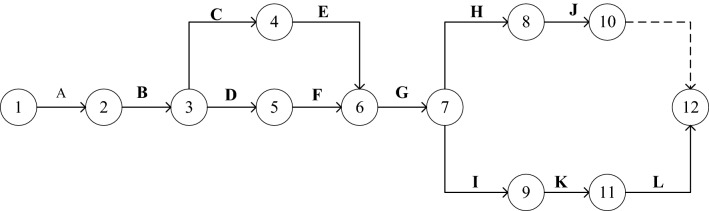
Two-code network plan for an engineering project.

Tianlun Central Seal project is located at the northeast corner of the intersection of Youth Road and Chaijiapo in Yueyanglou District. The site area of this project is 8845.99 square meters. Building 1 of this project is a 1$+$25F-story residential building with elevated first floor and property use; Building 2 is a 1$+$22F-story residential building with commercial use on the first floor; Building 3 is a 6-story apartment building with commercial use on the first floor and hotel-style apartments on the second to fifth floors. The study selects the assembled building 1 as the research object, with frame shear structure and prefabricated components including exterior wall panels, interior wall panels and laminated panels, etc. The prefabrication rate reaches 55$\%$. This project has 12 processes and a duration of 320 days, with a total project completion bonus of 10,000 yuan/day and a delay claim of 20,000 yuan/day. Its network plan is shown in Fig. 4; the contractor conducts a comprehensive study of the construction environment on site, collects and calculates the harmful pollutant emissions after considering the environmental level of the project in Table 2, and the corresponding emission factors of each pollutant in Table 3; the list of carbon emission factors that may be involved in the project is shown in Table 4. The relevant data and parameters of each process are shown in Table 5 (where${\alpha _{2i}}$=6 ,${\alpha _{4i}}$=0.05, based on the empirical method to determine the weight coefficients, the importance of the three optimization objectives in the sustainable goal in decreasing order, that is, take ${\omega _1}$=0.4, ${\omega _2}$=0.3, ${\omega _3}$=0.3.)

**Table 2:** Emissions of common hazardous pollutants.

Work process activities	Air pollutants			Solid waste			Sewage		
	*CO*	$SO{_2}$	$NO{_X}$	Cement	Dirt	Dust	*TSS*	*N*	$E{I_i}$
A	1294.9	2471.8	8789.5	123.6	648.5	3474.8	20988.32	14826.1	60.6
B	2536.1	3478.9	7987.5	548.9	365.2	4412.3	26253.1	6954.5	58.9
C	412.3	998.7	11028.9	158.6	398.4	8457.4	3441.25	24563.2	45.6
D	10569.3	498.6	1789.5	132.8	297.5	10547.1	3884.5	20045.3	49.0
E	4079.6	7892.4	4771.5	145.9	129.1	3198.6	40136.5	3562.4	59.0
F	10784.3	934.5	731.5	256.4	473.1	2341.8	14896.2	4256.9	78.6
G	1018.3	10089.6	2987.4	142.7	464.2	3103.7	3221.5	50934.2	51.4
H	10463.5	1047.3	4701.3	563.9	697.6	5472.3	14563.2	36595.2	63.2
I	6089.1	2986.4	3479.8	229.6	189.4	3058.9	3945.7	50241.6	65.6
J	227.1	47891.2	5029.1	356.2	148.6	6231.7	20441.5	7541.6	37.4
K	3298.5	10071.6	3996.5	125.9	1109.6	1789.4	11435.6	47589.3	67.8
L	4478.3	5021.8	7469.3	4478.3	784.3	5871.2	20635.2	3712.7	61.7

**Table 3:** List of pollutant emission factors^37–43^.

Pollutant category	Pollutant category	Emission factors	Literature sources
Air pollutants	*CO*	0.87	
	SO$_2$	0.45	^37–^^39^
	NO$_X$	0.15	
	Waste cement	0.29	
Solid waste	Abandoned dirt	0.70	^40,41^
	Dust	0.26	
Sewage	*TSS*	1.7	^42,43^
	*N*	2.4

**Table 4:** Inventory of carbon emission factors^20,44–46^.

Sources of carbon emissions	Type	Carbon emission factors	Data source
People	Workers breathing	1.72kg CO$_2$/(Man/working day)	^20^
Materials	Concrete (C30)	321.3kg CO$_2$eq/m$^3$	^44,45^
	Hot rolled steel bars (carbon steel)	2340 kg CO$_2$eq/t	
	Steel plate	2702 kg CO$_2$eq/t	
	Coatings	12 kg CO$_2$eq/t	
	ACC blocks	23 0 kg CO$_2$ eq/m$^3$	
Equipment	Electricity	1.04 kg CO$_2$/(KW h)	^45,46^
	Gasoline	2.26 kg CO$_2$/kg	
	Diesel	2.73 kg CO$_2$/kg

**Table 5:** Algorithm-related parameters.

Work sequence	${t_{si}}$	${t_{ni}}$	${t_{li}}$	${\alpha _{1i}}$	${C_{ni}}$	${C_{Ti}}$	$W{I_i}$
A	44	46	47	2.4	198	55.05	45
B	38	43	57	3.1	185	45.15	39
C	67	71	74	6.1	240	65.25	54
D	39	41	45	1.7	175	49.65	37
E	34	36	49	5.0	160	43.95	33
F	29	34	38	2.8	144	35.05	28
G	19	25	27	4.0	120	26.55	26
H	18	22	24	3.6	116	24.15	17
I	28	31	33	4.8	135	36.45	27
J	12	20	25	5.1	102	12.35	19
K	34	39	46	2.9	165	46.55	43
L	32	35	39	3.1	158	41.05	41

### NSGA-II algorithm parameter setting and operation

**Figure 5:**
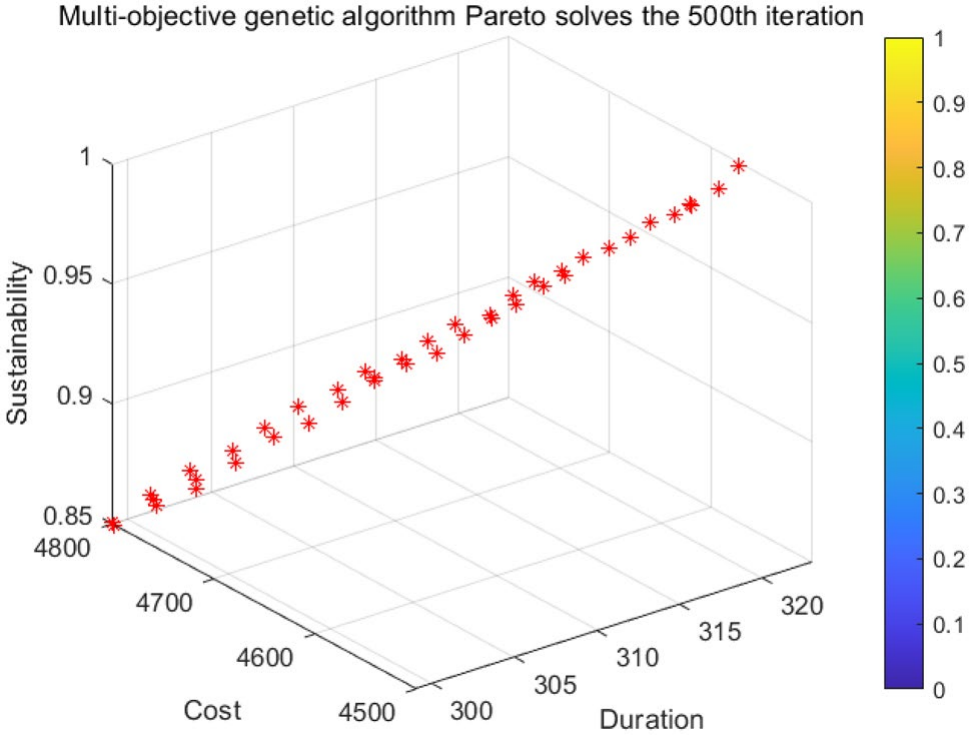
Schedule-cost-sustainable Pareto frontiers.

The relevant data were entered into the NSGA-II algorithm model and calculated using MATLAB R2021b software. To ensure the rationality of the parameters and the validity of the results, the parameters in the algorithm: the initial population is taken as 60, the number of evolution is taken as 500, the crossover factor is 0.9, and the probability of variation is 0.1. The program was run on a PC configured with an Intel COREi5 processor with 6 GB of RAM, and each run time was 10 minutes to end. The results of the computations are shown in Table 6, and only some of the Pareto solutions are listed in Table 6 due to the limitation of space; the optimal Pareto frontier diagram is shown in Fig. 5.

**Table 6:** Target optimal value and corresponding solution.

Programs	T	C	K
1	298	4834.46	0.84
2	299	4797.86	0.85
3	299	4813.36	0.85
4	299	4803.84	0.85
5	300	4783.26	0.87
6	300	4772.36	0.86
7	300	4777.96	0.86
8	301	4751.26	0.88
9	301	4759.46	0.88
$\vdots$	$\vdots$	$\vdots$	$\vdots$

### Analysis of results

**Figure 6:**
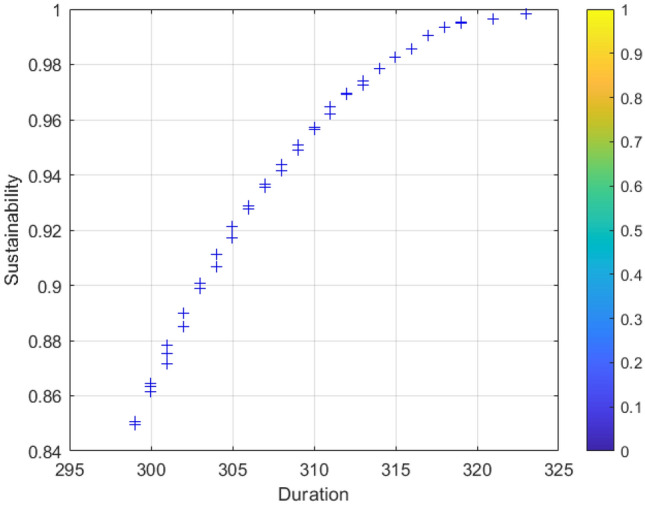
Sustainable-duration balance analysis.

**Figure 7:**
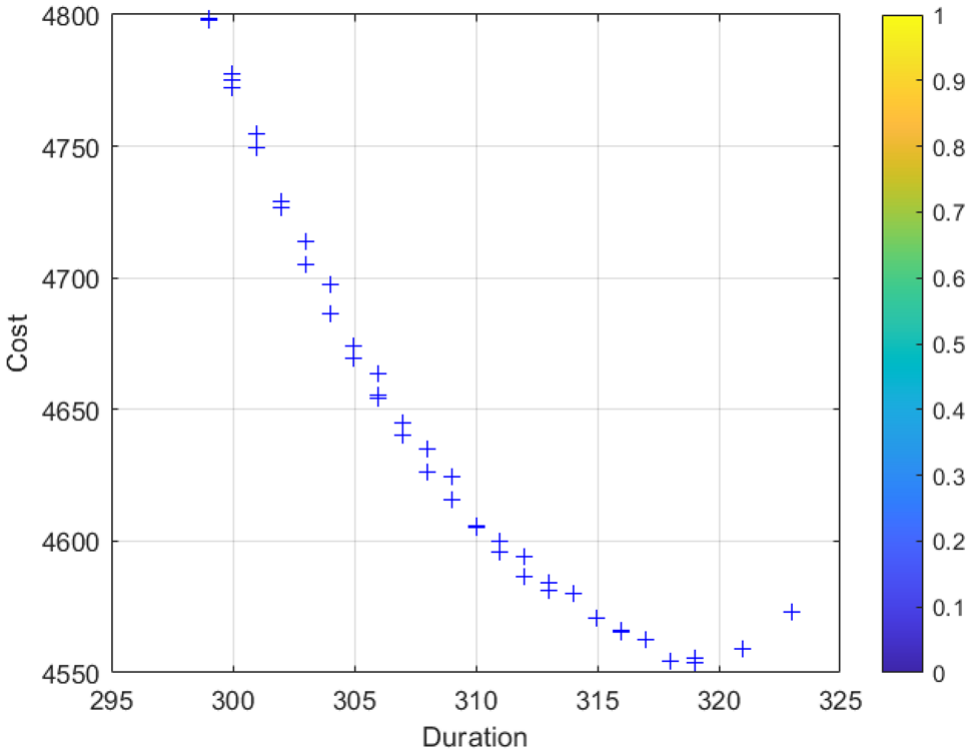
Cost-duration balance analysis.

**Figure 8:**
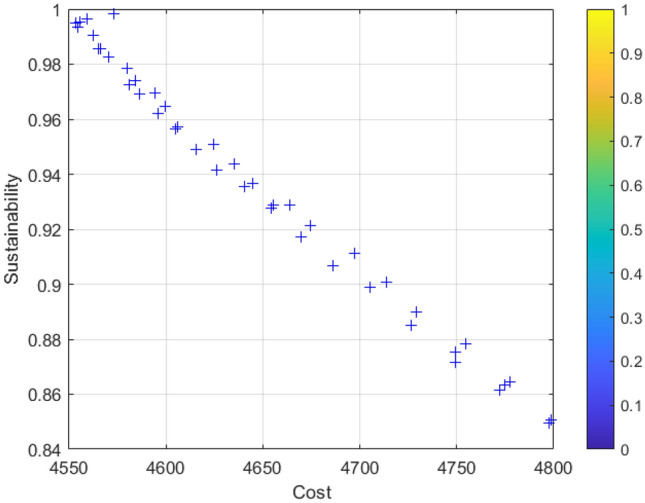
Sustainable-cost balance analysis.

**Figure 9:**
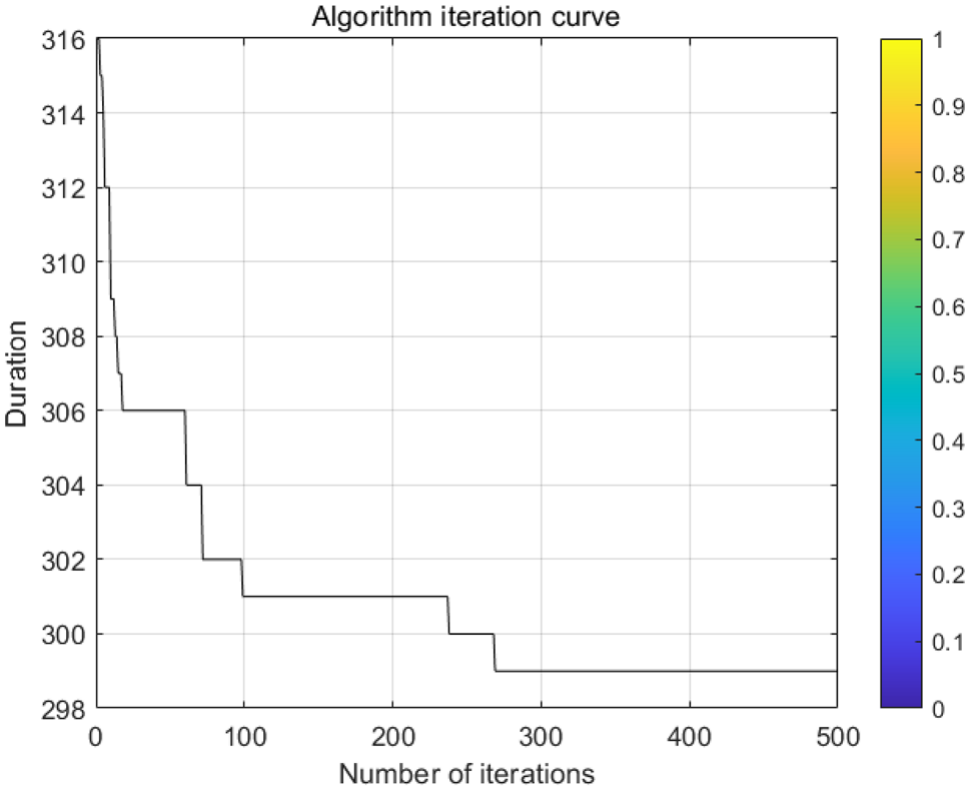
Duration iteration.

**Figure 10:**
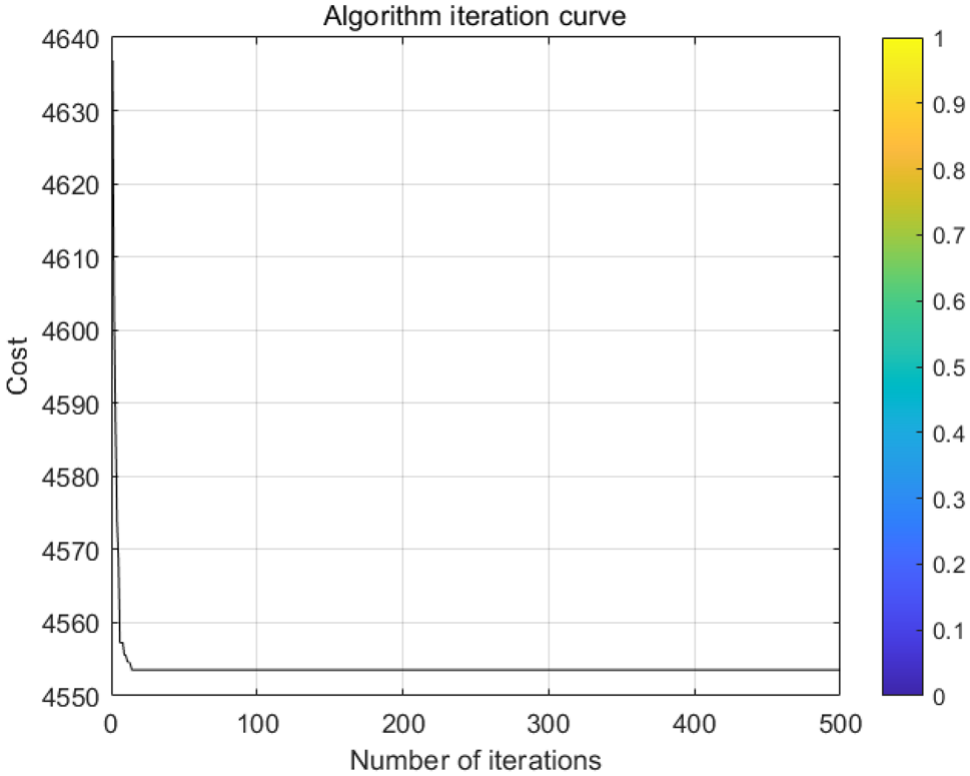
Cost iteration.

**Figure 11:**
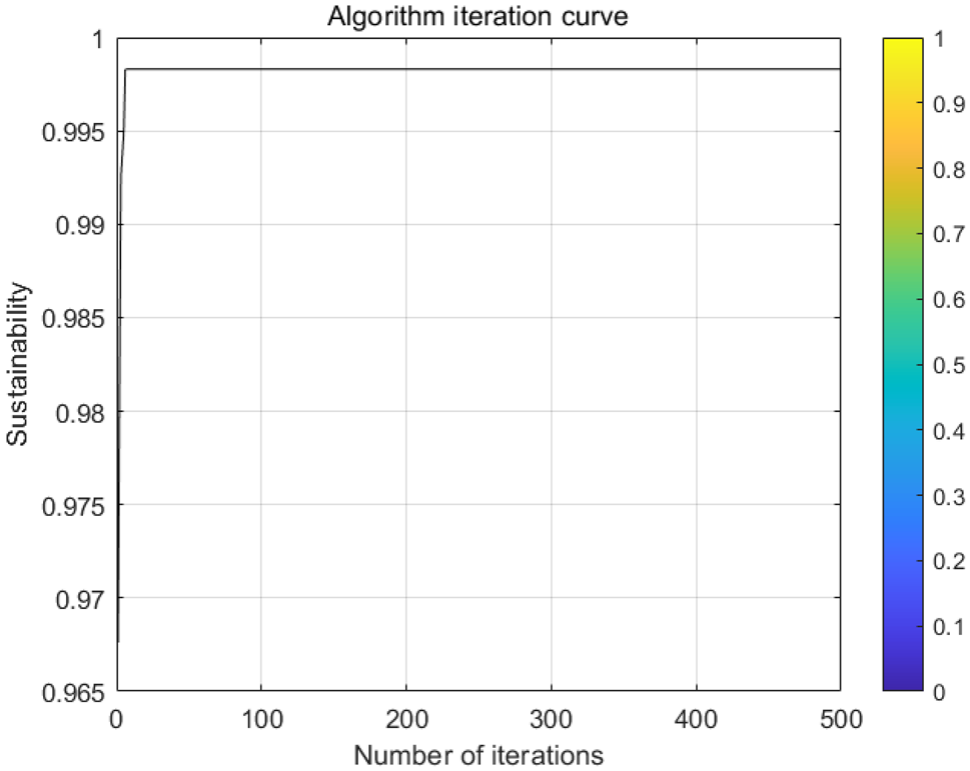
Sustainable iteration.

Each set of Pareto solutions can be used as an alternative for the project. After optimization, the shortest duration of the project is 298 days, which is 22 days shorter than the contract duration, and the longest duration is 301 days, which is 19 days shorter than the contract duration. and the two-dimensional relationship between sustainability and duration, cost and duration, and sustainability and cost, respectively. Figure 6 shows that as the cost of construction increases, the economic benefits of the construction project gradually decreases leading to an overall decrease in its sustainability level; Fig. 7 shows that as the duration is extended in a reasonable range, more jobs can be provided to ensure its social benefits leading to an overall increase in its sustainability level; Fig. 8 shows that the cost will increase due to the shortening of the duration. Figures 9, 10 and 11 show that with the iteration of the algorithm, the duration target gradually decreases and finally reaches the shortest duration 298; the cost target is unstable in the 0th–50th iterations, and gradually stabilizes and stays in the range of 4550–4560 in the 50th–500th iterations; the sustainability level target is unstable before the 50th iteration, and then gradually increases and remain stable. Therefore, to achieve sustainable maximization during the construction process, we need to reasonably control the construction period, consider the construction period, cost and sustainability as a system, coordinate and balance repeatedly, organize the construction reasonably, and strive to achieve the optimum of the whole target system.

### Program decision analysis

In practice, the most satisfactory alternative is selected by giving priority to a certain objective in project decision-making according to the nature of the project, optimizing resource allocation for the implementation of each process activity and enhancing economic efficiency. They are mainly divided into the following cases.

1. Projects that prioritize the guaranteed duration. Such projects require the shorter the duration the better, such as rescue and relief, post-disaster reconstruction projects, the builder needs to quickly reflect and complete the task in a very short period of time. Often these projects are implemented under resource tolerance conditions and are special measures taken by the state to deal with special situations. For such projects, the project decision maker can choose the one with shorter construction period from a series of Pareto optimal solutions as the actual construction plan.
2. Works that prioritize cost control. This kind of project requires to reduce the project cost as much as possible, save investment and get more profit by controlling the cost, such as affordable housing. For such projects, the project decision maker can choose the lower cost solution from a series of Pareto optimal solutions as the actual construction solution.
3. Priority is given to projects that focus on sustainable levels. Such projects require maximum resource saving, environmental pollution reduction and sustainability level improvement, such as green construction projects and municipal energy-saving projects. For such projects, the project decision maker can choose the solution with higher sustainability level from a series of Pareto optimal solutions as the actual construction solution. For simplicity, this paper adopts the efficacy coefficient method to make decision on the scheme.

For simplicity, this paper adopts the efficacy factor method to make decisions on the options. Since the duration and cost are cost-based attribute indicators and the sustainability level index is a benefit-based indicator, the positive ideal solution for this case is${A^ + }$ (298,4560.1,0.99) and the negative ideal solution${A^ - }$ (320,4834.5,0.84) according to the optimization results, and the efficacy coefficients of some Pareto solutions are shown in Table 7.

15 $$\begin{aligned}{} & {} \mathrm{{Benefit-based metrics:}} \,\, {\mathrm{{x}}_{{{\rm i}}}} {{ = }}\frac{{{y_i} - \min {y_i}}}{{\max {y_i} - \min {y_i}}} \end{aligned}$$

16 $$\begin{aligned}{} & {} \mathrm{{Cost - based metrics:}} \,\, {x_i} = \frac{{\max {y_i} - {y_i}}}{{\max {y_i} - \min {y_i}}} \end{aligned}$$

17 $$\begin{aligned} {\delta _i} = \root 3 \of {{{x_T}{x_C}{x_Q}}} \end{aligned}$$

**Table 7:** Partial Pareto solution efficacy factor.

Programs	${x_T}$	${x_C}$	${x_Q}$	$\delta$
1	1.000	0.000	0.000	0.000
2	0.955	0.134	0.067	0.204
3	0.955	0.077	0.067	0.170
4	0.955	0.112	0.067	0.192
5	0.909	0.187	0.200	0.324
6	0.909	0.226	0.133	0.302
7	0.909	0.206	0.133	0.292
8	0.864	0.303	0.267	0.412
9	0.864	0.273	0.267	0.398

In summary, it is obvious that the larger ${\delta _i}$ is, the better the design solution is, and it can be concluded that the optimal solution in Table 6 is solution 7. The duration of each process of solution 8 is shown in Table 8.

**Table 8:** Program 8 Duration of each process.

Programs	A	B	C	D	E	F	G	H	I	J	K	L
8	44	38	69	40	34	33	21	21	28	19	35	32

## Discussion

In this study, a duration-cost-sustainability level multi-objective optimization model is constructed with the duration of each job in the two-code network diagram as the independent variable. In order to solve the model effectively, a series of Pareto-optimal solutions are obtained using NSGA-II algorithm. The optimization results show that there is a mutually constraining relationship among the three objectives, which fits the engineering reality, i.e., the duration is shortened, the cost is increased, and the sustainability level is subsequently reduced; thus, the decision maker can choose the implementation plan that meets the requirements in conjunction with the actual engineering situation. Based on this, this study adopts the efficacy coefficient method for solution decision making, and the results show that the Pareto solution set can provide effective support for project managers’ decision making.

### Contrast analysis

This multi-objective optimization study on assembled buildings is different from the previous traditional multi-objective optimization studies on engineering projects. Analyzed from the perspective of multi-objective optimization of engineering projects, most studies focus on project duration, cost and quality. In recent years, more and more scholars have started to focus on green and low-carbon buildings, and Liang^20^ used an ant colony algorithm to optimize the cost-duration-carbon emission model of assembly building processes and obtained different combinations of process execution modes. Few scholars have introduced sustainability objectives into the traditional multi-objective equilibrium optimization of engineering management to provide decision makers with the best construction alternatives.From the perspective of research on the sustainability aspects of assembled building projects, most scholars have analyzed the sustainability evaluation of assembled buildings from a life-cycle perspective, Onat et al.^25^ evaluated the sustainability of residential buildings in the United States by using the LCSA framework, Hossaini et al.^47^, Nt analyzed two cases of six-story structural systems in Canada by using the sustainability evaluation framework for high-rise residential buildings Some scholars have also analyzed the sustainability of assembled buildings from the perspective of supply chain management, and Hu^30^ conducted a scientific evaluation study on the sustainability of assembled buildings in terms of designers, manufacturers, and suppliers in the supply chain of assembled buildings. However, most of the research on it stays in qualitative analysis, combining the analysis of assembled building sustainability with multi-objective management, and the quantitative research on assembled is even more lacking. Based on this, this paper quantitatively analyzes the economic, environmental and social impacts of assembled buildings from the perspective of sustainability, followed by adding sustainability objectives into the traditional multi-objective integrated optimization of assembled buildings, and finally solving the duration-cost-sustainability model by algorithms to provide a reference for the sustainable development of assembled buildings.

### Advantages and limitations

This study draws on the previous research results and has the following two improvements; first, considering the carbon emission cost, the total cost model including the minimum carbon emission cost is established for the carbon emission problem generated during the construction of the assembly building, which is in line with the green concept of the construction industry. Secondly, it innovatively introduces sustainability objectives into the traditional multi-objective equilibrium optimization of engineering management, and uses NSGA-II algorithm to solve the multi-objective optimization model by combining with engineering project examples. The sample examples of Pareto optimal solutions and the comparison of results obtained by the model calculation provide decision makers with optimal solutions within the available decision range. As a result, the decision maker can select the implementation solution that meets the requirements in conjunction with the actual engineering situation. This optimization idea can realize the optimal allocation of construction resources and has practical value for scientific and sustainable development research of assembled buildings. However, the model still has some limitations: in the process of quantifying the sustainability goals, the optimal quantification method has not been determined, more dimensions of the sustainability goals have not been analyzed, and the influence of some secondary factors on the sustainability goals has been ignored. Further research is still needed on how to build an optimization model that is more applicable to such difficult-to-quantify goals as sustainability in the future.

### Reliability and universality

This study provides project decision makers with optimization ideas for adding sustainability requirements to traditional objective management, which can be applied to assembly building projects in different countries. In order to highlight the multi-objective optimization management of assembly buildings under the perspective of sustainable construction, limited by space, etc., the study is conducted only from the dynamic process of assembly building construction. Based on the multi-objective optimization model of cost-duration-sustainability level of construction projects constructed in this study, construction companies can collate the actual engineering parameters of the project and bring them into the model, and subsequently apply artificial intelligence algorithms to solve its multi-objective problems, and project decision makers can select suitable alternatives based on each set of Pareto solutions, providing references for the sustainable development of local assembled buildings.

### Research significance

The building industry is a major contributor to global energy consumption and carbon emissions, and the implementation of sustainable building brings environmental, economic and social benefits at all levels. It is widely believed that sustainable construction is the basic path and process to achieve sustainable development goals and green buildings. Introducing sustainable goals into engineering project management multi-objective optimization is a new type of management concept. It refers to the activity of improving the economic and social benefits of the project and minimizing the consumption of resources and the lowest negative impact on the environment through scientific management and technology within a reasonable construction period. It has theoretical significance to the development of local sustainable construction.

The multi-objective optimization model and solution algorithm proposed in this study can be applied to guide engineering practice, and the introduction of sustainability objectives into traditional engineering project management can improve the sustainability of assembled buildings, provide project decision makers with the best construction alternatives according to the specific requirements and different preferences of different projects in terms of duration, cost and sustainability level, and make the comprehensive optimization management of engineering projects after considering sustainability level more It is suitable for modern management optimization.

## Conclusions

In this paper, the sustainability objective is introduced into the duration-cost integrated optimization model, and a multi-objective integrated optimization model of duration-cost-sustainability level of engineering projects is constructed. This study draws on the previous research results and has the following two improvements; first, it considers carbon emission cost and establishes a total cost model including the minimum carbon emission cost for the carbon emission problem generated during the construction of the assembly building, which is in line with the green concept of the construction industry. Secondly, it innovatively introduces sustainability objectives into the traditional multi-objective equilibrium optimization of engineering management, and uses NSGA-II algorithm to solve the multi-objective optimization model by combining with engineering project examples. The sample examples of Pareto optimal solutions and the comparison of results obtained by the model calculation provide decision makers with optimal solutions within the available decision range. As a result, the decision maker can select the implementation plan that meets the requirements in conjunction with the actual engineering situation. After optimization, the shortest duration of the project is 298 d, which is 22 d shorter than the contract duration; the longest duration is 301 d, which is 19 d shorter than the contract duration, resulting in 5.66$\%$ cost reduction and 6.87$\%$ duration reduction, and the efficacy factor method is applied to find option 8 as the optimal option among the alternatives. In summary, this optimization idea can realize the optimal allocation of construction resources and has practical value for scientific and sustainable development research of assembled buildings. However, the model still has some limitations: in the process of quantifying the sustainability goals, the optimal quantification method has not been determined, more dimensions of the sustainability goals have not been analyzed, and the influence of some secondary factors on the sustainability goals has been ignored. Further research is still needed on how to build an optimization model that is more applicable to such difficult-to-quantify goals as sustainability in the future.

## Data Availability

The data underlying the results presented in the study are included within the manuscript.
